# Alien Hand, Restless Brain: Salience Network and Interhemispheric Connectivity Disruption Parallel Emergence and Extinction of Diagonistic Dyspraxia

**DOI:** 10.3389/fnhum.2016.00307

**Published:** 2016-06-20

**Authors:** Ben Ridley, Marion Beltramone, Jonathan Wirsich, Arnaud Le Troter, Eve Tramoni, Sandrine Aubert, Sophie Achard, Jean-Philippe Ranjeva, Maxime Guye, Olivier Felician

**Affiliations:** ^1^Aix-Marseille Université, CNRS, CRMBM UMR 7339Marseille, France; ^2^APHM, Hôpitaux de la Timone, CEMEREMMarseille, France; ^3^APHM, Hôpitaux de la Timone, Service de Neurologie et NeuropsychologieMarseille, France; ^4^Aix Marseille Université, Inserm, INS, Institut de Neurosciences des SystèmesMarseille, France; ^5^AP–HM, Hôpitaux de la Timone & Hôpital Henri-Gastaut, Service de Neurophysiologie CliniqueMarseille, France; ^6^GIPSA-Lab F-38000, University Grenoble AlpesGrenoble, France; ^7^GIPSA-Lab, F-38000, Centre National de la Recherche Scientifique (CNRS)Grenoble, France

**Keywords:** alien hand, callosal agenesis, disconnection syndrome, graph theory, resting-state, functional connectivity, epilepsy

## Abstract

Diagonistic dyspraxia (DD) is by far the most spectacular manifestation reported by sufferers of acute corpus callosum (CC) injury (so-called “split-brain”). In this form of alien hand syndrome, one hand acts at cross purposes with the other “against the patient’s will”. Although recent models view DD as a disorder of motor control, there is still little information regarding its neural underpinnings, due to widespread connectivity changes produced by CC insult, and the obstacle that non-volitional movements represent for task-based functional neuroimaging studies. Here, we studied patient AM, the first report of DD in patient with complete developmental CC agenesis. This unique case also offers the opportunity to study the resting-state connectomics of DD in the absence of diffuse changes subsequent to CC injury or surgery. AM developed DD following status epilepticus (SE) which resolved over a 2-year period. Whole brain functional connectivity (FC) was compared (Crawford-Howell [CH]) to 16 controls during the period of acute DD symptoms (Time 1) and after remission (Time 2). Whole brain graph theoretical models were also constructed and topological efficiency examined. At Time 1, disrupted FC was observed in inter-hemispheric and intra-hemispheric right edges, involving frontal superior and midline structures. Graph analysis indicated disruption of the efficiency of salience and right frontoparietal (FP) networks. At Time 2, after remission of diagnostic dyspraxia symptoms, FC and salience network changes had resolved. In sum, longitudinal analysis of connectivity in AM indicates that DD behaviors could result from disruption of systems that support the experience and control of volitional movements and the ability to generate appropriate behavioral responses to salient stimuli. This also raises the possibility that changes to large-scale functional architecture revealed by resting-state functional magnetic resonance imaging (fMRI) (rs-fMRI) may provide relevant information on the evolution of behavioral syndromes in addition to that provided by structural and task-based functional imaging.

## Introduction

Acute damage to the corpus callosum (CC) may generate a cluster of clear-cut inter-hemispheric disconnection (IHD) “split-brain” symptoms reflecting the inability to convey sensory information to contralateral motor or linguistic output areas (Tomasch, 1954; Sperry, 1968a). In rare cases it may also produce diagonistic dyspraxia (DD; Akelaitis, 1945), a fascinating neuropsychological syndrome involving involuntary inter-manual conflicts where one hand acts at cross purposes with the other. DD has remained the most representative “alien hand” syndrome, a term encompassing a variety of seemingly goal-directed movements occurring after a brain insult, performed without the intention of the actor (Scepkowski and Cronin-Golomb, 2003; Biran and Chatterjee, 2004; Berlucchi, 2012). Although DD has been observed in cases of acquired CC damage, to our knowledge it has never been described in developmentally-based CC dysgenesis.

Agenesis of the CC (AgCC), occurring in 1/4000 live births (Paul et al., 2007), may be partial or complete; may be found in isolation (primary AgCC) or as part of a wider developmental disorder (Paul et al., 2007); and is commonly associated with epilepsy (Taylor and David, 1998). In contrast to acquired lesions of the CC, primary AgCC has an overall limited and subtle impact on cognition and subjects with AgCC do not display clinically relevant IHD symptoms (Sperry, 1968b). To account for this puzzling fact—known as “Sperry’s paradox” (Sperry, 1968a)—compensatory structural pathways have been suggested. Neuroimaging has recently provided support for macrostructural changes in AgCC, evidencing intra- and interhemispheric white matter tracts providing bilateral links via the posterior and anterior commissures (Tovar-Moll et al., 2014). These are likely to compose, at least partly, the set of compensatory pathways preserving inter-hemispheric transfer. These observations are corroborated by a resting-state functional magnetic resonance imaging (rs-fMRI) study showing preserved functional connectivity (FC) between homotopic cortices in AgCC subjects (Tyszka et al., 2011). Additionally, intrinsic connectivity networks (ICNs) in the AgCC group were similar to those identified in controls, suggesting that global functional architecture remains largely preserved in AgCC. Paralleling the clinically-defined Sperry’s paradox, these findings stand in sharp contrast with the drastic disruption of inter-hemispheric FC observed following surgical interruption of the CC (Johnston et al., 2008).

Here, we report on an epileptic AgCC patient who developed pervasive DD behaviors following status epilepticus (SE), with gradual improvement over 2-years. Patient AM offers a rare opportunity to longitudinally study the functional underpinning of DD, without the major impact produced by acute callosal section/lesions on connectomics. By examining rs-fMRI connectivity via edgewise comparison of FC and graph theoretical modeling, we demonstrate large scale disorganization and reorganization paralleling the emergence and extinction of IHD and DD symptoms.

## Case Report

### Patient History

Patient AM (45 years at first scan) is an ambidextrous man with a long-standing history of complex partial seizures in the setting of mesial temporal lobe epilepsy. AM was first referred to our center in 2011. He had been treated by carbamazepine (800 mg/day) and phenobarbital (100 mg/day) over the past two decades and partial seizures had occurred on a monthly basis. Awake EEG demonstrated left temporal slowing and spikes. Brain MRI revealed left hippocampal sclerosis, along with complete AgCC and posterior commissure, but an intact anterior commissure. A first neuropsychological assessment was undertaken in September 2011. On the Wechsler Adult Intelligence Scale-III (WAIS-III), he demonstrated low to average global cognitive functioning (full IQ = 74), with mild dissociation between verbal and performance scales (respectively VIQ = 80 and PIQ = 70).

In March 2012, AM was admitted for SE of unknown duration, that left him with a right-sided motor weakness (“Todd’s paralysis”) that resolved over a 2-week period. Routine structural brain MRI was unchanged. Lamotrigine was added to his previous medication regimen (up to 400 mg/day). One month later, and despite full resolution of epileptic seizures, he started noticing a series of embarrassing and distressing behaviors, described as unpredictable and uncontrollable, and occurring on a daily basis. The most relevant examples being: (i) walking in his neighborhood, his right hand would suddenly grab a corner post rendering him unable to turn; AM would then remain stuck to the post for several minutes, desperately turning around, until his hand permitted disengagement; he would then trap his right hand in his jacket to keep walking; (ii) while driving his car, his right hand would suddenly turn the steering wheel to the left, despite intending to make a right-turn; he observed similar behaviors with his right foot, which would maintain pressure on the accelerator pedal whilst intending to slow down; (iii) while putting on his trousers with his left hand, his right hand would pull them off; the same behaviors could also occur while attempting to don a pair of socks or a sweat shirt, and several times for each piece of clothing; and (iv) his right hand would “play tricks” on him, spiriting away his wallet from the back pocket of his pants, and refusing to give it back to him.

### Neuropsychological Evaluations

AM underwent a detailed assessment in September 2012 (6 months after SE onset). Full IQ was 69, with stable verbal IQ (VIQ = 80) but slightly decreased performance IQ (PIQ = 63). While the DD symptoms constituted the main interferent with AM’s quality of life, additional neuropsychological testing revealed further subtle manifestations of IHD. Right-sided constructional apraxia, right ideomotor apraxia and right visual anomia were evidenced in addition to the right-handed DD described above. Only rightward constructional apraxia is concordant with expectations of IHD in right-handed subjects, and the pattern of deficits in Patient AM suggests a bi-hemispheric organization of language, gestural and constructional skills, with right hemisphere prevalence. With the exception of enduring ideomotor problems in terms of tool-use pantomimes with his right hand, follow-up indicated significant amelioration (September 2013) and extinction (September 2014) of all neuropsychological symptoms (see Supplementary Figure 1). In parallel, DD behaviors were reported as very occasional in September 2013 and had totally resolved over the 6 months preceding the final evaluation in September 2014. At this time, awake EEG was within normal range aside from occasional left temporal slowing and spikes (see Supplementary Figure 2).

## Procedure

### MRI Acquisition

Patient AM was scanned in December 2012 (Time 1—acute phase) and February 2015 (Time 2—remission). Sixteen control (male, right-handed, mean age: 41 ± 16 years) cross-sectional data-sets were used for comparison. All controls underwent a pre-scan medical interview and had no history of neurological or psychiatric illness, substance abuse or psychotropic medication. Participants gave informed consent to take part in this study, with local Ethics Committee approval (Comité de Protection des Personnes Sud Méditerranée 1).

A 3T Verio scanner (Siemens, Erlangen, Germany) with a 12 channel receiver coil was used to obtain structural high resolution T1-weighted images magnetisation-prepared rapid gradient-echo (MP-RAGE); repetition time (TR) = 1900 ms, echo time (TE) = 2.2 ms, inversion time (TI) = 900 ms, 1 × 1 × 1 mm voxels) and 15 min of functional gradient echo planar images (250 volumes, TR = 3600 ms, TE = 28 ms, flip angle = 90°, 50 axial slices interleaved, 2.5 × 2.5 mm, 122 × 122 matrix). Participants were asked to close their eyes and not to think about anything in particular in a taskless “resting-state” condition.

### fMRI Processing and Graph Construction

For a full description of processing and graph construction please see Ridley et al. (2015). Briefly, after realignment and slice-timing (SPM8, Wellcome Trust Centre, London, UK), regional masks in subject-space (FLIRT, FMRIB Software Library^1^) based on the AAL template (Tzourio-Mazoyer et al., 2002) were used to obtain functional time-series from which nuisance regressors obtained from regions of interest (ROIs) in white matter and ventricles and motion parameters (three planes) were removed.

A wavelet transform (MODWT) was used to obtain the range 0.035–0.07 Hz (wavelet scale 2; Achard et al., 2006). The resulting data was used to populate an 84 × 84 correlation matrix. Each cell—or edge—in this correlation matrix represents the Pearson correlation of wavelet coefficient time courses between two non-cerebellar regions of the AAL, and allowed us to compare the strength of correlation between each set of two regions in Patient AM vs. the same regional pairs in controls in an edgewise fashion.

We additionally applied graph theoretical analysis, a complementary technique which can provide information on multiscale, parallel and distributed features of patterns of connectivity (Sporns, 2014). For each individual, three adjacency matrices were created with the highest 10% (349), 20% (697), and 30% (1046) significant FDR-controlled Pearson correlation coefficients between regions, which were used to construct unweighted brain graphs. Degree as well as global and local efficiency were derived using* Brainwaver* (Version 3.0.2, The R Foundation for Statistical Computing; for detailed review see Rubinov and Sporns, 2010). Briefly, degree is the number of edges connecting to a node and is a broad measure of it importance/centrality (Bernhardt et al., 2013). Efficiency is the inversion of the mean shortest path length (*L*_i,j_) between a given node and a given set of other nodes (Onias et al., 2014), including the rest of the network (Global Efficiency, E_glob_) or just the immediate neighbors of a node (Local Efficiency, E_loc_). In healthy cortex a balance between a region’s ability to subserve its specialized function in collaboration with related structures in the immediate neighborhood (segregation/local integration) and its ability to integrate long-range information from distributed regions (global integration) is thought to be optimal (Achard and Bullmore, 2007).

### Control Comparison Sample and Statistical Methodology

**Figure 1:**
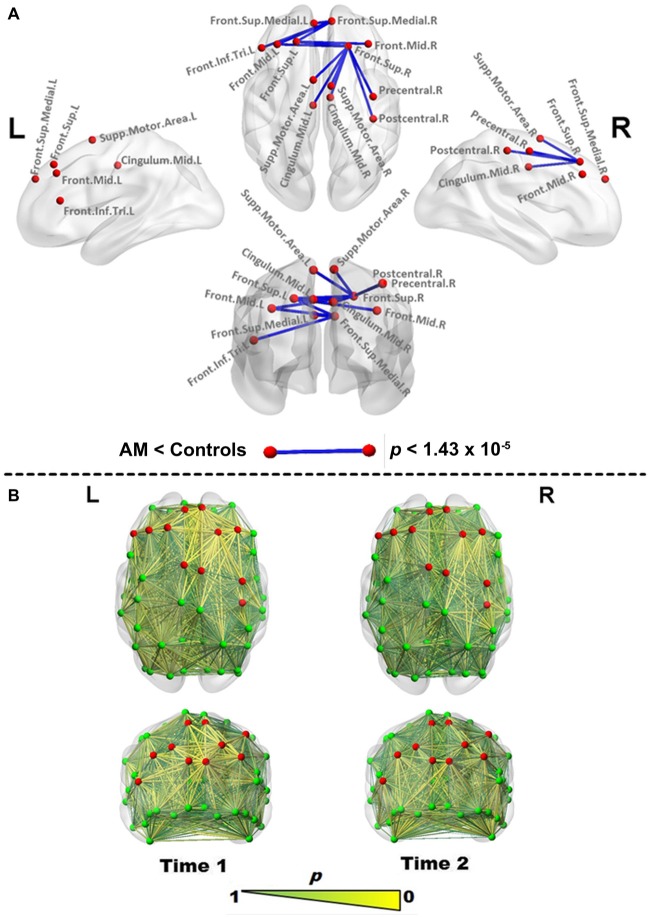
**Edgewise functional connectivity (FC) changes in Patient AM. (A)** Significantly reduced edges (blue lines) in Patient AM compared to controls at Time 1 using the Crawford-Howell (CH) test. Differences were considered significant at a Bonferroni-corrected level of* p* < 1.43 × 10^−5^. Red nodes are used to highlight the regions joined by a significantly changed link, and are reproduced for comparison in all figures. **(B)** Abnormality map at Time 1 (left) and Time 2 (right), making use of the *p* value generated by the CH test as a point estimate of abnormality to scale edges in size and color (larger, more yellow being more abnormal), indicating the proportion of controls with FC values as extreme as those found for each edge in the patient. Note the bilateral frontal normalization vs. retention of frontoparietal (FP) abnormalities at Time 2. Abbreviations used: Sup, superior; Mid, Middle; Inf, inferior; Supp, Supplementary; Tri, triangular; L, Left; R, Right. Brain networks were visualized with the BrainNet Viewer (Xia et al., 2013).

To compare AM to controls we used the Crawford-Howell (CH) modified two-tailed *t*-test specifically designed for case-control comparisons (Crawford and Garthwaite, 2012). The CH-test evaluates the null hypothesis that a case is drawn from the control distribution and the *p*-value additionally serves as a point-estimate of abnormality of the patient’s score, indicating the proportion of controls more extreme, and both applications were utilized here (see Figure 1). See Crawford and Garthwaite (2012) for formal proof of this dual role, and Monte Carlo simulations demonstrating better control of Type I errors and greater robustness to violations of normality than other case-control approaches. Edgewise comparisons were Bonferroni-corrected at *p* < 1.43 × 10^−5^.

Graph theoretical metrics were also compared at whole brain and hemispheric scales as well as in 10 canonical ICNs. See Supplementary Figure 3 for network partitions.

The CH-test was also used to confirm Patient AM and controls did not differ in terms of age and motion parameters (Supplementary Information 1).

## Results

### Edgewise FC

During the acute phase of DD symptoms (Time 1), edgewise analysis indicated a network of significantly reduced (Bonferroni-corrected) interhemispheric connections and intrahemispheric connections in the right (but not left) hemisphere (Figure 1A, Table 1), which abated at the second scan (Time 2). Only a single edge between the left middle and inferior occipital gyri (*t*_(15)_ = −8, *p* < 1.43 × 10^−5^) was found to be significantly reduced relative to controls.

**Table 1:** **Significantly reduced edges in Patient AM vs. controls at Time 1**.

Node 1	Node 2	*t*	FC (R): Patient	FC (R): Cont. Mean (± SD)
Frontal_Sup_L	Frontal_Sup_R	−14.08	0.41	0.91 (± 0.03)
	Frontal_Mid_R	−6.42	0.60	0.87 (± 0.04)
	Frontal_Sup_Medial_R	−6.87	0.19	0.82 (± 0.09)
Frontal_Sup_R	Frontal_Mid_L	−8.01	0.37	0.85 (± 0.06)
	Precentral_R	−7.33	0.34	0.86 (± 0.07)
	Supp_Motor_Area_L	−11.28	0.27	0.87 (± 0.05)
	Supp_Motor_Area_R	−7.26	0.19	0.85 (± 0.09)
	Cingulum_Mid_L	−8.02	0.26	0.86 (± 0.07)
	Cingulum_Mid_R	−7.04	0.31	0.86 (± 0.08)
	Postcentral_R	−6.82	0.35	0.83 (± 0.07)
Frontal_Sup_	Frontal_Mid_L	−6.66	0.16	0.78 (± 0.09)
Medial_R				
	Frontal_Inf_Tri_L	−6.55	0.03	0.70 (± 0.1)
	Frontal_Sup_Medial_L	−11.28	0.49	0.91 (± 0.04)

*Differences were considered significant at a Bonferroni corrected level of p < 1.43 × 10^−5^. Abbreviations used: FC, functional connectivity; Cont.; Control; SD, standard deviation; Sup, superior; Mid, Middle; Inf, inferior; Supp, Supplementary; Tri, triangular; L, Left; R, Right*.

The *p*-value yielded by the CH test was also used in its role as a point estimate of abnormality, as depicted in Figure 1B. At Time 1, prominent areas of abnormality are indicated fronto-centrally in both hemispheres and fronto-parietally in the right hemisphere. At Time 2, the scan demonstrates the spatial stability of these areas of abnormality, while indicating substantial “improvement”.

### Graph Theoretical Analysis

Results from graph analysis (Table 2) at Time 1 indicate disturbances to E_glob_, E_loc_ and in the average number of edges (degree) within the right hemisphere. At Time 2, global and local efficiency disturbances have abated, though enduring degree differences are evident at some sparsities.

**Table 2:** **Scales/Networks showing significant differences in graph theoretical indices**.

	E_glob_					E_loc_					Degree				
Scale/Network	Sparsity		10	20	30	Sparsity		10	20	30	Sparsity		10	20	30
**Right hemisphere**	C. Mean		0.32	0.48	0.58	C. Mean		0.61	0.75	0.81	C. Mean		9.25	18.5	27.4
	C. SD		0.04	0.04	0.04	C. SD		0.08	0.06	0.05	C. SD		0.74	1.19	1.57
	T1	Pat	0.23	0.39	0.51	T1	Pat	0.48	0.64	0.7	T1	Pat	8.32	16.1	25.8
		*t*	−3.1	−2.9	−3.1		*t*	−1.2	−3.2	−2.9		*t*	−3.4	−4.5	−4.7
		*p*	**<0.01**	**0.01**	**0.01**		*p*	**0.24**	**0.01**	**0.01**		*p*	**<0.01**	**<0.01**	**<0.01**
	T2	Pat	0.26	0.45	0.56	T2	Pat	0.49	0.68	0.74	T2	Pat	8.26	16.3	23.9
		*t*	−1.9	−0.6	−0.2		*t*	−0.7	−0.5	−0.3		*t*	−0.4	−2.4	−2.3
		*p*	0.08	0.54	0.78		*p*	0.51	0.64	0.71		*p*	0.69	**0.03**	**0.03**
**Frontoparietal right**	C. Mean		0.36	0.53	0.63	C. Mean		0.63	0.79	0.84	C. Mean		9.05	20.3	31.2
	C. SD		0.05	0.05	0.05	C. SD		0.13	0.05	0.04	C. SD		2.31	3.8	5.03
	T1	Pat	0.11	0.23	0.36	T1	Pat	0.10	0.53	0.43	T1	Pat	4.43	7.86	13.00
		*t*	−4.8	−6.3	−5.8		*t*	−4.1	−4.9	−8.8		*t*	−2.00	−3.2	−3.5
		*p*	**<0.01**	**<0.01**	**<0.01**		*p*	**<0.01**	**<0.01**	**<0.01**		*p*	0.07	**0.01**	**<0.01**
	T2	Pat	0.14	0.42	0.53	T2	Pat	0.34	0.63	0.71	T2	Pat	2.29	9.86	17.9
		*t*	−4.2	−2.3	−2.8		*t*	−2.3	−2.9	−2.8		*t*	−2.3	−2.9	−2.8
		*p*	**<0.01**	**0.03**	**0.04**		*p*	**0.04**	**0.01**	**0.01**		*p*	**0.04**	**0.01**	**0.01**
**Salience**	C. Mean		0.35	0.51	0.61	C. Mean		0.65	0.77	0.82	C. Mean		9.98	20.2	29.5
	C. SD		0.08	0.08	0.08	C. SD		0.16	0.08	0.08	C. SD		3.31	5.29	6.64
	T1	Pat	0.17	0.28	0.39	T1	Pat	0.44	0.51	0.53	T1	Pat	5.33	11.4	16.6
		*t*	−2.3	−2.6	−2.6		*t*	−1.3	−3.3	−3.6		*t*	−1.4	−1.6	−1.9
		*p*	**0.04**	**0.02**	**0.02**		*p*	**0.21**	**0.01**	**<0.01**		*p*	0.19	0.13	0.08
	T2	Pat	0.24	0.45	0.56	T2	Pat	0.42	0.8	0.86	T2	Pat	5.42	13.3	21.9
		*t*	−1.3	−0.7	−0.6		*t*	−1.4	0.31	0.44		*t*	−1.3	−1.3	−1.1
		*p*	0.21	0.49	0.58		*p*	0.18	0.76	0.67		*p*	0.2	0.22	0.28

*Differences were considered significant at p < 0.05 (indicated in bold type). Abbreviations used: C, control; SD, Standard deviation; Pat, Patient; T1, Time 1; T2; Time 2*.

**Figure 2:**
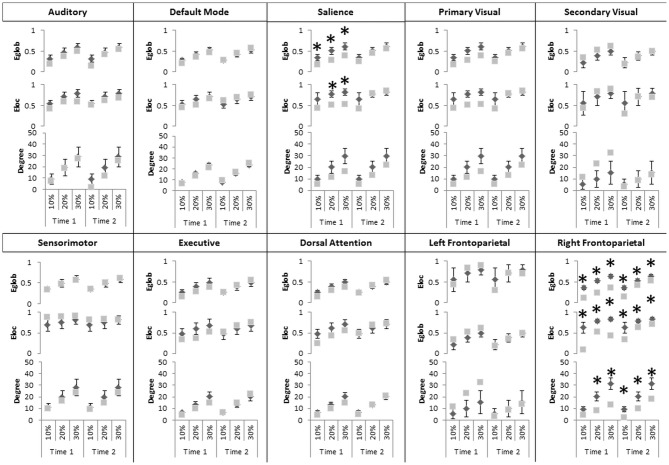
**Significant changes in graph theoretical indices in intrinsic connectivity networks (ICNs).** Differences, considered significant at *p* < 0.05 indicated by black asterisks, at Time 1 and Time 2 for the topological efficiency of connections between nodes and the entire brain network (E_glob_ top), with immediate neighbors (E_loc_, middle) and the number of edges connecting to a node (Degree, bottom). Gray squares represent graph indices in AM and dark diamonds represent mean graph indices in controls. X-axis indicates connection sparsity as a percentage of all possible connections. Note that y-axis minima and maxima are different across metrics. Abbreviations used: Aud, Auditory; DMN, Default Mode Network, SM, Sensorimotor; Vis, Visual; Prime, Primary; Sec, Secondary; FP, Frontoparietal; Dors, Dorsal; Atten, Attention.

At the Intrinsic Connectivity Network scale (Figure 2, Table 2) at Time 1, all metrics were disrupted within the right fronto-parietal network, and E_glob_ and E_loc_ within the salience network. At Time 2, the salience network had recovered in terms of topological efficiency and no longer exhibited significantly extreme graph metric estimates relative to controls while the right fronto-parietal network demonstrated enduring disturbances.

## Discussion

Patient AM represents a unique case of DD in a developmentally acallosal patient, highlighting a novel complication of comorbid AgCC and epilepsy, as well as serving as a test case for the use of resting-state analyses in a little-understood disorder.

According to recent theoretical accounts, “alien” movements occur because affordances supplied by the environment reflexively generate motor primitives that are not inhibited by an intended action (Frith et al., 2000; Biran et al., 2006). The patient is aware that an action is produced (via sensory feedback), but the actual action is performed despite no intention and no postulation (feed-forward intention) of a predicted bodily change. Failure of inhibitory signals arising from the supplementary motor area (SMA) acting on a sensorimotor (SM) fronto-parietal system involved in the production and coordination of motor primitives has also been posited, either because of defective inhibition (e.g., lesions of the SMA) or an impairment of its inter-hemispheric transfer (e.g., lesions of the CC; Frith et al., 2000; Biran et al., 2006). Other accounts stress a form of “split-brain” phenomena: movements are purposeful and reasonable given each hemisphere’s competences, but the “intention” is inaccessible and thus “alien” due to failed interhemispheric integration (Verleger et al., 2011).

Activation-fMRI studies have attempted to delineate the neural basis of alien hand syndromes (Assal et al., 2007; Schaefer et al., 2010). Collectively, they suggest that both intentional and non-intentional movements involve a common set of regions, principally primary motor, supplementary motor and pre-motor areas, but differ in the activation of the inferior frontal gyrus (IFG). However, conclusions regarding the role of the IFG could not be drawn, since the activation studies are in apparent disagreement: associating the IFG with either intentional (Assal et al., 2007) or non-intentional movements (Schaefer et al., 2010).

Given its minimal demands on the individual being scanned, a resting-state approach offers an adjunct to traditional imaging approaches that need not rely on specific symptoms or tasks that may be difficult in DD (Scepkowski and Cronin-Golomb, 2003; Berlucchi, 2012). Our results (Figure 1A) implicate inter-hemispheric disruption between a set of frontal regions identified in the above activation studies (Assal et al., 2007; Schaefer et al., 2010) in both intentional and non-intentional movements (superior mesial, pre-central and SMA regions), as well as those that distinguish them (i.e., the IFG). Additionally, graph analysis implicates reduced efficiency in the salience network (Figure 2). The salience network is a recently described large-scale network that comprises frontoinsular regions and the anterior cingulate cortex (ACC), along with limbic and subcortical structures (Seeley et al., 2007). Comparisons between intrinsic connectivity and task-based co-activation derived from many thousands of functional datasets indicate the salience network encompasses a wide range of processes including integration of bodily-related information and conflict/error monitoring (Smith et al., 2009), positioning it remarkably well for the transitional role between extero-/interoception and cognition originally posited by Seeley et al. (2007): as a sensory integrator and filter capable of “tagging” information as relevant or irrelevant for higher-level “executive” networks. Concordantly, salience network disruption observed in frontotemporal dementia is thought to be specifically implicated in disinhibited behaviors (Farb et al., 2013; Zhou and Seeley, 2014). Thus, our results may reflect a disruption of a system involved in the ability to select the relevant sensory stimuli, and/or inhibit non-intended actions.

Furthermore, patients experience non-volitional movements as not only unplanned but also as distinctly “other” or “alien” (Scepkowski and Cronin-Golomb, 2003; Biran and Chatterjee, 2004; Biran et al., 2006). Both edgewise and ICN results indicate disruption of an inter-hemispheric network that task-based neuroimaging implicates in distinguishing self/other-generated actions, including dorsomedial frontal cortices and one of the salience network’s key nodes (the insular region; Farrer and Frith, 2002; Farrer et al., 2003; Sperduti et al., 2011). Interestingly, corticobasal degeneration can also result in volitional experience and movement aberrations falling under the rubric of “alien hand” (c.f. Schaefer et al., 2016). In a group of patients suffering from this condition, Wolpe et al. (2014) revealed modified FC of the pre-SMA with a set of fronto-parietal regions that included the anterior component of the salience network.

Taken together, the normalization of connectivity and network organization with remission of DD may reflect a regained ability to detect and disregard irrelevant SM programs primed by environmental affordances, and a restored sense of authorship in self-generated actions. Enduring modifications within a right hemisphere could reflect remaining difficulties in tool-use pantomimes (Supplementary Figure 1), consistent with the role of FP regions in complex actions relying on stored information such as tool use (Wheaton and Hallett, 2007).

We suggest that disconnection symptoms and DD were likely prompted by SE, unbalancing the inter-hemispheric transfer that may have previously transited via an intact anterior commissure which subserved the patient’s previously “normal” behavioral profile (Franz, 2012; Winter and Franz, 2014). Comorbities in patient AM, while potential sources of vulnerability seem unlikely to account for the acute onset and trajectory described here. Ongoing seizure activity cannot explain AM’s DD, since seizures have fully resolved. Despite distributed network effects of epilepsy (Bettus et al., 2009, 2010; Guye et al., 2010; Ridley et al., 2015), changes involving the regions identified here are for the most part identified in children and young adults and represent developmental and non-acute processes (Ibrahim et al., 2014; Luo et al., 2014; Li et al., 2015; Wei et al., 2015). Likewise, despite drastic and life-long structural modifications (Kasprian et al., 2013; Jakab et al., 2015) AgCC it is not equivalent to callosotomy (Owen et al., 2013), and recent evidence suggests acallosal brains can support a largely “normal” cognitive and intrinsic FC repertoire in the form of preserved homotopic connectivity and bilaterally symmetric ICN architecture (Tyszka et al., 2011; Tovar-Moll et al., 2014). This conclusion is bolstered by the fact that partitioning at gross anatomical scales (Supplementary Figure 4) does not yield equivalent graph metric changes, underlining the relevance of the functional network partitions employed here.

However, despite the forgoing, larger homogenous samples—including separate longitudinal control, AgCC and epilepsy patient groups—are certainly necessary to address the limitations of this study and confirm Patient AM as a general model of alien hand syndromes. Furthermore, the test-retest reliability of connectomics metrics places a lower limit on the interpretability of data, though the stability of our data over time as suggested by Figure 1B militate against this being the main determinant of our results. Future work will also need to speak to known sources of variance in connectomics, including age, gender, handedness, hemispheric dominance, medication, and vigilance state (Bettus et al., 2009; Liu et al., 2009; Tian et al., 2011; Vlooswijk et al., 2011; Tagliazucchi and Laufs, 2014).

## Conclusion

Our data suggest that the salience network and interhemispheric connectivity play a role in supporting the experience and control of volitional movements and the ability to select the most relevant among internal and extrapersonal stimuli in order to produce appropriate behavior. Bearing methodological and sampling caveats in mind, the current work represents a promising first indication that rs-fMRI is relevant to the understanding of alien hand syndromes over time and, more broadly, of neuropsychiatric manifestations that may benefit in the same way from an adjunct to structural and task-based functional imaging. In this spirit, we hope that AM’s case will serve to marshal resources in pursuit of a better understanding of this difficult and rare disorder.

## Author Contributions

BR, OF, MG, J-PR, MB and JW contributed to the design of the work, acquisition analysis and interpretation of data. ALeT, ET, SaA and SoA contributed to the acquisition, analysis and interpretation of data. All authors revised, contributed to and approved the final manuscript. All co-authors agree on all aspects of the work and ensure that questions related to the accuracy and integrity of any part of the submitted work are appropriately investigated and resolved.

## Funding

The funding sources (“PHRC-I 2013” EPI-SODIUM (grant number 2014-27)) had no involvement in study design; collection, analysis and interpretation of data; in the writing of the report; in the decision to submit the article for publication.

## Conflict of Interest Statement

The authors declare that the research was conducted in the absence of any commercial or financial relationships that could be construed as a potential conflict of interest.

## Footnotes

^1^www.fmrib.ox.ac.uk/fsl
