# Supplementary material for: Alien Hand, Restless Brain: Salience Network and Interhemispheric Connectivity Disruption Parallel Emergence and Extinction of Diagonistic Dyspraxia
Source: Front Hum Neurosci. 2016 Jun 20;10:307. doi: 10.3389/fnhum.2016.00307 (PMC4913492; doi:10.3389/fnhum.2016.00307)
Supplement: Supplementary file 1 [file DataSheet_1.docx]

Supplementary Material

Alien hand, restless brain: salience network and interhemispheric connectivity disruption parallel emergence and extinction of diagonistic dyspraxia

**Supplementary Info 1**

**Demographics**

The age of the patient was not significantly different (CH-Test) to controls at first (t (15)=0.24, *p=*0.8), or second scan (t (15)=0.35, *p=*0.73).

**Head motion**

Mean framewise displacement (mm) in 3 translational and rotational (degrees of arc converted to millimeters of displacement as per Power, Barnes, Snyder, Schlaggar, & Petersen (2012)) planes were not significantly different (CH-Test) between patient and controls at the first scan (*X*: t (15)=-0.25, *p=*0.8, *Y*: t (15)=-0.97, *p=*0.35, *Z*: t (15)=-1 *p=*0.33, *Pitch*: t (15)=-1, *p=*0.3, *Roll*: t (15)=-0.37, *p=*0.72), *Yaw*: t (15)=0.02, *p=*0.99) nor were they significantly different at the second scan (*X*: t (15)=1.1, *p=*0.29, *Y*: t (15)=-0.61, *p=*0.55, *Z*: t (15)=0.32 *p=*0.75, *Pitch*: t (15)=-0.34, *p=*0.74, *Roll*: t (15)=1.07, *p=*0.3), *Yaw*: t (15)=0.06, *p=*0.96).

Power, J. D., Barnes, K. A., Snyder, A. Z., Schlaggar, B. L., and Petersen, S. E. (2012). Spurious but systematic correlations in functional connectivity MRI networks arise from subject motion. *NeuroImage* 59, 2142–2154. doi:10.1016/j.neuroimage.2011.10.018.
