# Supplementary material for: Alien Hand, Restless Brain: Salience Network and Interhemispheric Connectivity Disruption Parallel Emergence and Extinction of Diagonistic Dyspraxia
Source: Front Hum Neurosci. 2016 Jun 20;10:307. doi: 10.3389/fnhum.2016.00307 (PMC4913492; doi:10.3389/fnhum.2016.00307)
Supplement: Supplementary file 2 [file Image_1.pdf]

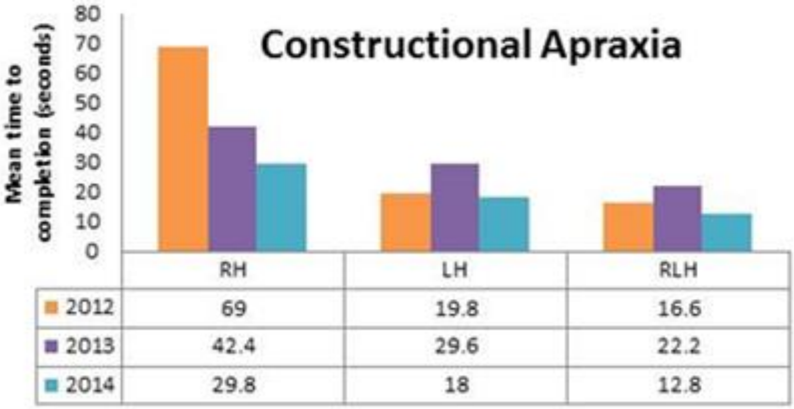

	RH	LH	RLH
2012	69	19.8	16.6
2013	42.4	29.6	22.2
2014	29.8	18	12.8

Constructional tasks involve reproducing shapes using set of plastic triangles. Performed using the right hand (RH), the left hand (LH) and both hands (RLH), and scores correspond to the mean time in seconds to completion across shapes.

Patient asked to produce pantomimes, symbolic gestures and tool-use gestures from sets numbering as indicated in brackets. Numbers indicate the amount of errors within each set.

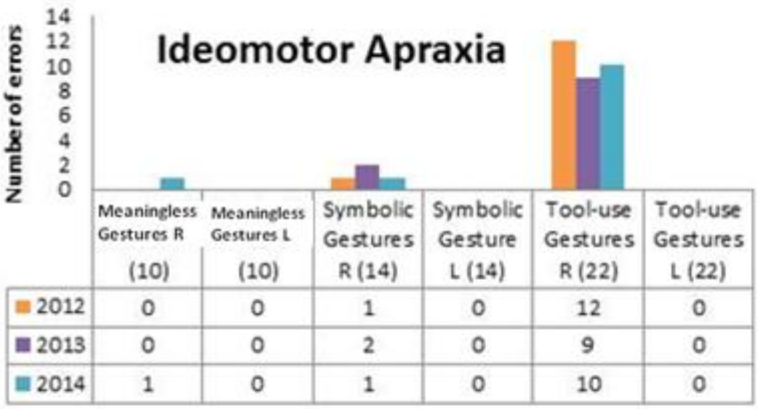

	Meaningless Gestures R (10)	Meaningless Gestures L (10)	Symbolic Gestures R (14)	Symbolic Gesture L (14)	Tool-use Gestures R (22)	Tool-use Gestures L (22)
2012	0	0	1	0	12	0
2013	0	0	2	0	9	0
2014	1	0	1	0	10	0

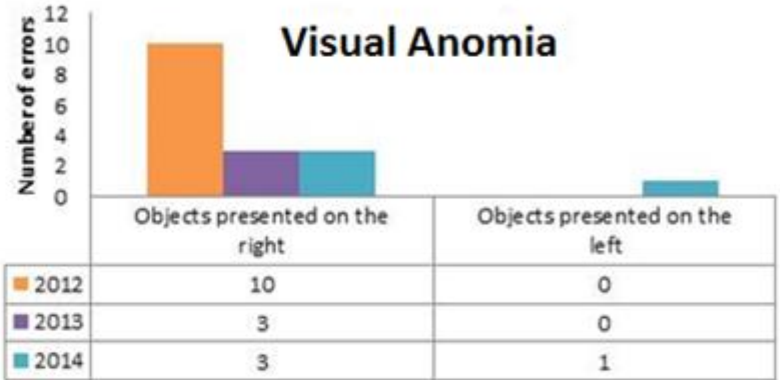

	Objects presented on the right	Objects presented on the left
2012	10	0
2013	3	0
2014	3	1

Test measures number of errors in naming objects presented to the left and right visual field only.

Supplementary Figure 1: Results from neuropsychological follow-up across three years in AM. Focusing on inter-hemispheric transfer of information, neuropsychological testing included : 1) single word writing upon verbal command with either hand; 2) constructional abilities, with either hand and the simultaneous use of both hands, evaluated with the block design subtest of the WAIS-III and the construction of a pyramid-like geometric figure composed of 15 identical cubes; 3) praxis with either hand, using a battery composed of 14 symbolic gestures, 22 pantomimes of tool use and 10 meaningless gestures; 4) reading capacity with 30 words, matched for length and frequency, tachiscopically and randomly presented in either visual hemi-field; 5) naming capacity for 30 pictures of common objects and living entities, tachiscopically and randomly presented in either visual hemi-field; 6) naming abilities of 10 common and holdable objects placed in either hand while blindfolded; 7) dichotic listening of 32 words pairs, matched for length and frequency. Note that the right side, in addition to diagnostic dyspraxia, is disproportionately affected by constructional apraxia and visual anomia, and that this ameliorates over time. The main indicator of ideomotor apraxia is difficulties with tool-use pantomimes with the right hand, and this issue endured in 2014 despite remission of the other IHD symptoms. Performance at dichotic listening test was within normal range (data not shown). No tactile anomia was detected when AM was required to name and identify various objects placed in either hand. The patient showed no evidence of agraphia with either hand. At first evaluation (2012) however, writing revealed diagonistic behaviors, his right hand suddenly refusing to keep writing and crossing off the several words that had just been written.
