# Supplementary material for: Alien Hand, Restless Brain: Salience Network and Interhemispheric Connectivity Disruption Parallel Emergence and Extinction of Diagonistic Dyspraxia
Source: Front Hum Neurosci. 2016 Jun 20;10:307. doi: 10.3389/fnhum.2016.00307 (PMC4913492; doi:10.3389/fnhum.2016.00307)
Supplement: Supplementary file 3 [file Image_2.pdf]

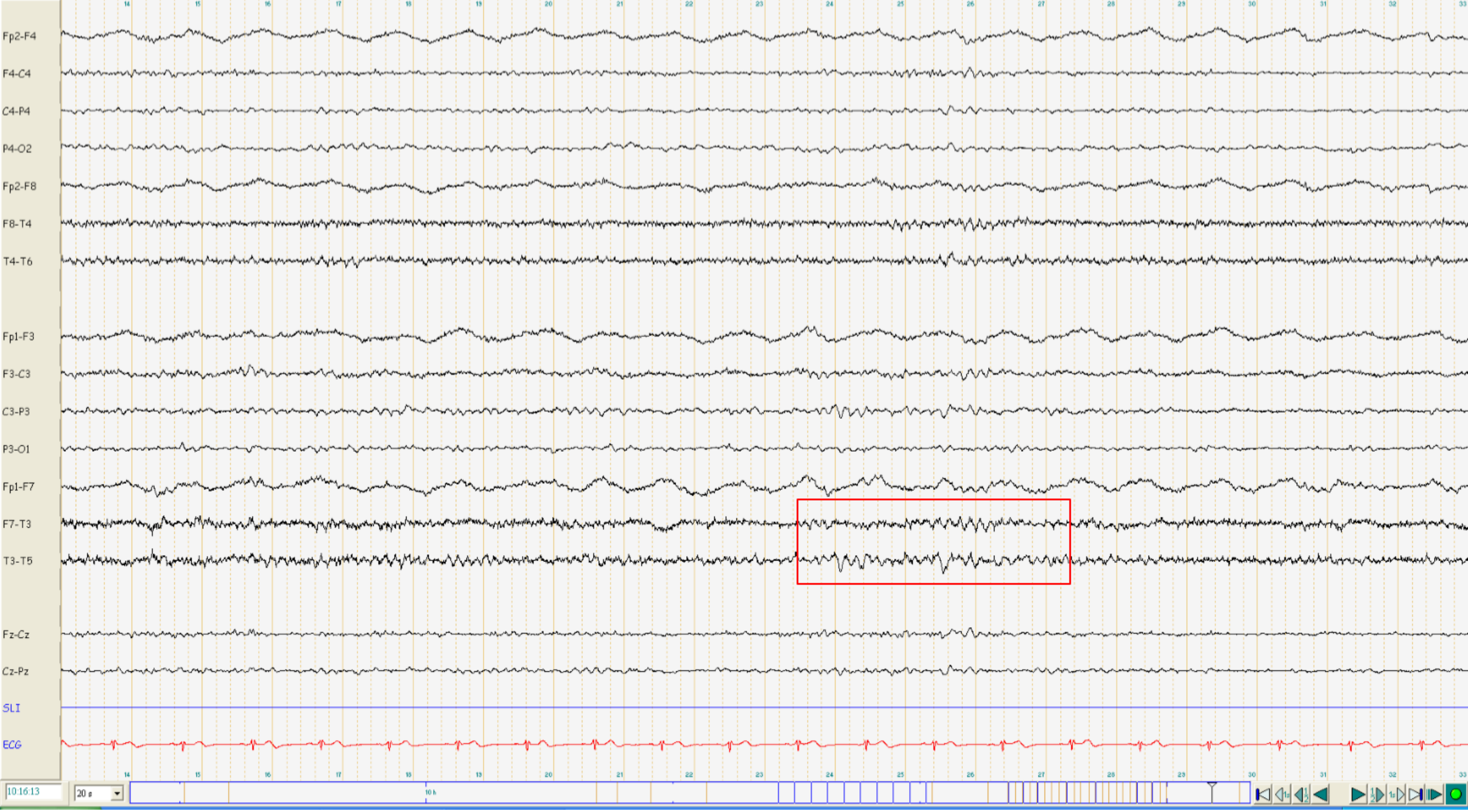

Supplementary Figure 2: Awake EEG performed at Time 2 (Remission). Minimal sharp and slow waves facing the left temporal electrodes (longitudinal bipolar montage; frontopolar regions are disrupted by sweat artifacts).
