# Supplementary material for: Alien Hand, Restless Brain: Salience Network and Interhemispheric Connectivity Disruption Parallel Emergence and Extinction of Diagonistic Dyspraxia
Source: Front Hum Neurosci. 2016 Jun 20;10:307. doi: 10.3389/fnhum.2016.00307 (PMC4913492; doi:10.3389/fnhum.2016.00307)
Supplement: Supplementary file 4 [file Image_3.pdf]

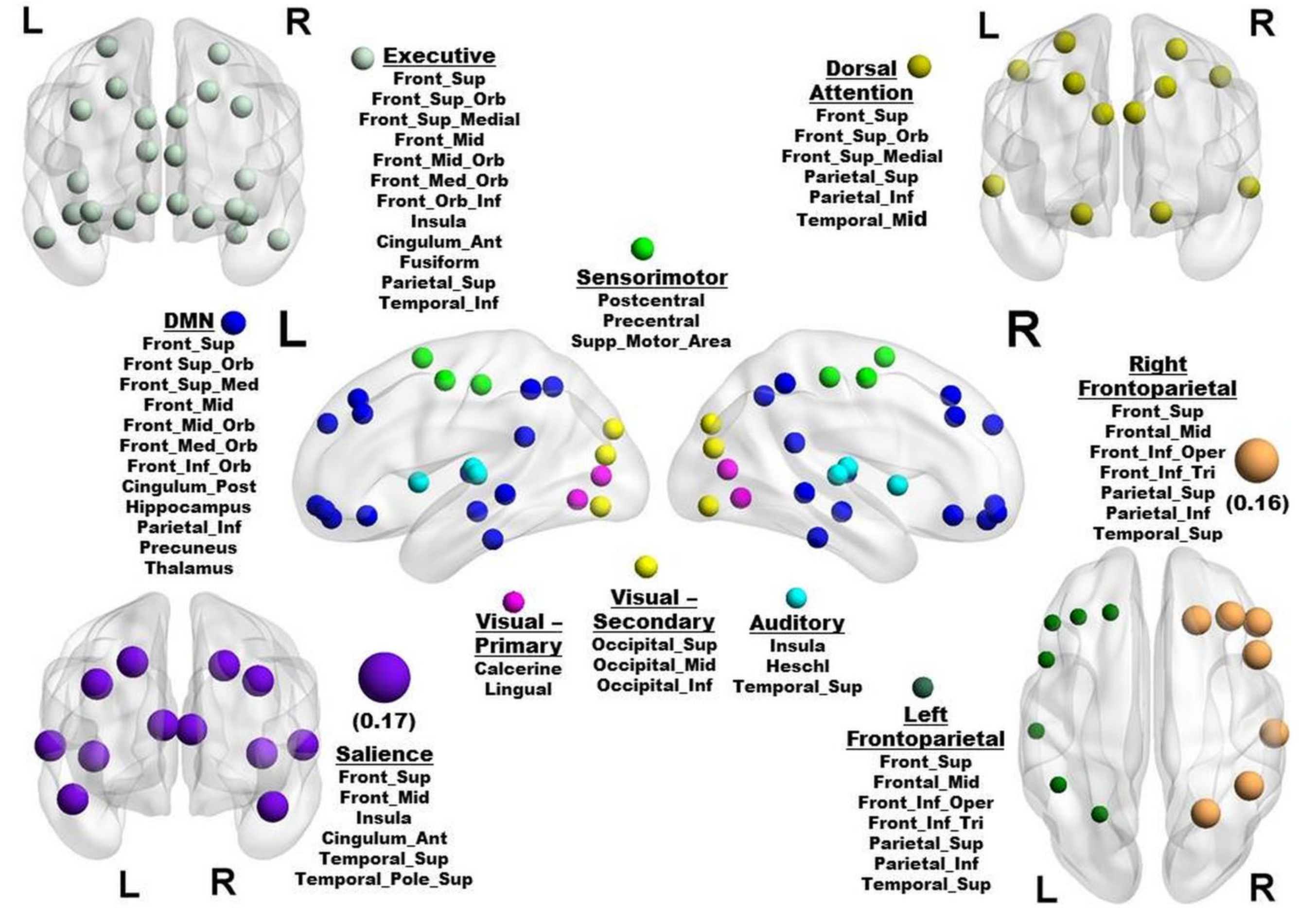

Figure 2: Schematic illustration of ICN node assignment and illustration of graph metric change in salience and right frontoparietal networks. Nodes belonging to ten ICNs are depicted using spheres centred on MNI-space centroids. As the only networks that showed significant differences with controls at Time 1, spheres for salience and right frontoparietal networks have been scaled for illustrative purposes in proportion to the change in E_{glob} (ΔE_{glob}) over time (Time2-Time1), as calculated for a connection sparsity of 30%. ΔE_{glob} for these networks is quantified in brackets on the figure. For reference all other networks, which failed to show significant differences at Time 1, have been set to an arbitrary size of 0.05. Abbreviations used: Sup, superior; Orb, Orbital; Mid, Middle; Inf, inferior; Ant, Anterior; Supp, Supplementary; Tri, triangular; L, Left; R, Right; DMN, Default Mode Network; Oper, Operculum. Brain networks were visualized with the BrainNet Viewer **[1]**.

Nodes were assigned to a given network based on a set of authoritative reviews **[2,3,4]** and where necessary the primary literature for certain networks: the auditory network ; Default Mode Network (DMN); sensorimotor, medial/primary and lateral/secondary visual networks; 2 symmetrical but unilateral frontoparietal networks (left and right); the salience, executive control and dorsal attention networks **[5,6,7]**.

[1] Xia, M., Wang, J., and He, Y. (2013). BrainNet Viewer: A Network Visualization Tool for Human Brain Connectomics. *PLoS ONE* 8. **[2]** van den Heuvel, M. P., & Hulshoff Pol, H. E. (2010). Exploring the brain network: a review on resting-state fMRI functional connectivity. European Neuropsychopharmacology 20(8), 519–534. **[3]** Raichle, M. E. (2011). The restless brain. *Brain Connectivity*, *1*(1), 3–12. **[4]** Smith, S. M., Fox, P. T., Miller, K. L., Glahn, D. C., Fox, P. M., Mackay, C. E., … Beckmann, C. F. (2009). Correspondence of the brain's functional architecture during activation and rest. *PNAS*, *106*(31), 13040–13045. **[5]** Fox, M. D., Corbetta, M., Snyder, A. Z., Vincent, J. L., & Raichle, M. E. (2006). Spontaneous neuronal activity distinguishes human dorsal and ventral attention systems. *PNAS*, *103*(26), 10046–10051. **[6]** Seeley, W. W., Menon, V., Schatzberg, A. F., Keller, J., Glover, G. H., Kenna, H., … Greicius, M. D. (2007). Dissociable intrinsic connectivity networks for salience processing and executive control. *The Journal of Neuroscience:*, *27*(9), 2349–2356. **[7]** Vincent, J. L., Kahn, I., Snyder, A. Z., Raichle, M. E., & Buckner, R. L. (2008). Evidence for a frontoparietal control system revealed by intrinsic functional connectivity. *Journal of Neurophysiology*, *100*(6), 3328–3342.
