# Supplementary material for: Alien Hand, Restless Brain: Salience Network and Interhemispheric Connectivity Disruption Parallel Emergence and Extinction of Diagonistic Dyspraxia
Source: Front Hum Neurosci. 2016 Jun 20;10:307. doi: 10.3389/fnhum.2016.00307 (PMC4913492; doi:10.3389/fnhum.2016.00307)
Supplement: Supplementary file 5 [file Image_4.pdf]

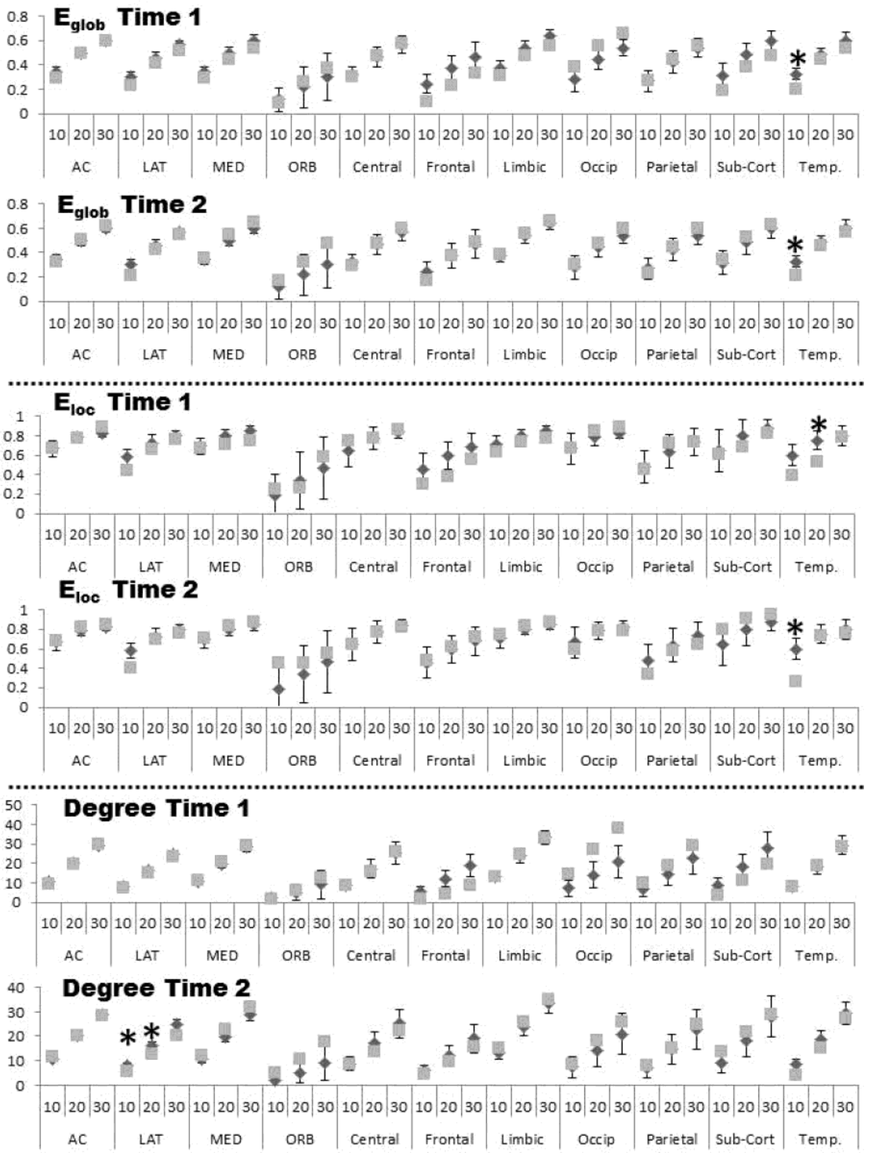

Supplementary Figure 3: Significant differences for a range of partitions other than ICNs. Differences were considered significant at P<0.05. Grey squares represent graph indices in AM and dark diamonds represent graph indices in controls. Given that Patient AM retains the anterior commissure (AC), which was presumably interrupted by his Status Epilepticus we investigated changes in regions with direct connections as indicated by diffusion tensor imaging **[1,2]** or post mortem dissection **[3]**. Given the possibility of gross morphological changes in the brains of patients with colossal agenesis, we investigated whether changes were localised to broadly-defined regions as a control against our contention that the changes in Patient AM are at the level of ICNs. The nodes included in each partition correspond to the labelling system in **[4]**. The sets of all lateral (LAT), medial (MED) and orbital (ORB) nodes were considered, as were all nodes within the Central, Frontal, Limbic, Occipital (Occip), Parietal and Temporal (Temp) lobes, and all sub-cortical (Sub-Cort) nodes. Note the lack of robust effects across all connection sparsities.

[1] Catani M, Howard RJ, Pajevic S, *et al.* Virtual in vivo interactive dissection of white matter fasciculi in the human brain. *NeuroImage* 2002;**17**:77–94. **[2]** Patel MD, Toussaint N, Charles-Edwards GD, *et al.* Distribution and fibre field similarity mapping of the human anterior commissure fibres by diffusion tensor imaging. *Magma N Y N* 2010;**23**:399–408. doi:10.1007/s10334-010-0201-3 **[3]** Di Virgilio G, Clarke S, Pizzolato G, *et al.* Cortical regions contributing to the anterior commissure in man. *Exp Brain Res* 1999;**124**:1–7. **[4]** Tzourio-Mazoyer N, Landeau B, Papathanassiou D, *et al.* Automated anatomical labeling of activations in SPM using a macroscopic anatomical parcellation of the MNI MRI single-subject brain. *NeuroImage* 2002;**15**:273–89. doi:10.1006/nimg.2001.0978
